# Mitochondria-associated programmed cell death: elucidating prognostic biomarkers, immune checkpoints, and therapeutic avenues in multiple myeloma

**DOI:** 10.3389/fimmu.2024.1448764

**Published:** 2024-12-11

**Authors:** Gongzhizi Gao, Jiyu Miao, Yachun Jia, Aili He

**Affiliations:** ^1^ Department of Hematology, The Second Affiliated Hospital of Xi’an Jiaotong University, Xi’an, China; ^2^ National-Local Joint Engineering Research Center of Biodiagnostics and Biotherapy, The Second Affiliated Hospital of Xi’an Jiaotong University, Xi’an, China; ^3^ Xi’an Key Laboratory of Hematological Diseases, Xi’an, China

**Keywords:** multiple myeloma, mitochondria, programmed cell death, prognosis genes, risk model

## Abstract

**Background:**

Multiple myeloma (MM) is a hematological malignancy characterized by the abnormal proliferation of plasma cells. Mitochondrial dysfunction and dysregulated programmed cell death (PCD) pathways have been implicated in MM pathogenesis. However, the precise roles of mitochondria-related genes (MRGs) and PCD-related genes (PCDRGs) in MM prognosis remain unclear.

**Methods:**

Transcriptomic data from MM patients and healthy controls were analyzed to identify differentially expressed genes (DEGs). Candidate genes were selected by intersecting DEGs with curated lists of MRGs and PCDRGs. Univariate Cox, least absolute shrinkage and selection operator (LASSO), multivariate Cox, and stepwise regression analyses identified prognostic genes among the candidates. A risk model was constructed from these genes, and patients were stratified into high- and low-risk groups for survival analysis. Independent prognostic factors were incorporated into a nomogram to predict MM patient outcomes. Model performance was evaluated using calibration curves, receiver operating characteristic (ROC) analysis, and decision curve analysis (DCA). Finally, associations between prognostic genes and immune cell infiltration/drug responses were explored.

**Results:**

2,192 DEGs were detected between MM and control samples. 30 candidate genes were identified at the intersection of DEGs, 1,136 MRGs, and 1,548 PCDRGs. *TRIAP1, TOMM7, PINK1, CHCHD10, PPIF, BCL2L1*, and *NDUFA13* were selected as prognostic genes. The risk model stratified patients into high- and low-risk groups with significantly different survival probabilities. Age, gender, ISS stage, and risk score were independent prognostic factors. The nomogram displayed good calibration and discriminative ability (AUC) in predicting survival, with clinical utility demonstrated by DCA. 9 immune cell types showed differential infiltration between MM and controls, with significant associations to risk scores and specific prognostic genes. 57 drugs, including nelarabine and vorinostat, were predicted to interact with the prognostic genes. Ultimately, qPCR in clinical samples from MM patients and healthy donors validated the expression levels of the seven key prognostic genes, corroborating the bioinformatic findings.

**Conclusion:**

Seven genes (*TRIAP1, TOMM7, PINK1, CHCHD10, PPIF, BCL2L1, NDUFA13*) involved in mitochondrial function and PCD pathways were identified as prognostic markers in MM. These findings provide insights into MM biology and prognosis, highlighting potential therapeutic targets.

## 1 Introduction

MM is a plasma cell malignancy characterized by clonal proliferation of neoplastic cells in the bone marrow. It is the second most prevalent hematological cancer, accounting for approximately 10% of all hematological malignancies. MM is frequently associated with devastating complications such as hypercalcemia, renal impairment, anemia, and bone lesions (1). MM progresses from an asymptomatic precursor stage - monoclonal gammopathy of undetermined significance (MGUS), to smoldering multiple myeloma (SMM), and finally to active MM (2). The etiology of MM remains incompletely elucidated, but it is hypothesized to involve a complex interplay between genetic predisposition, environmental factors, and immune system dysregulation. Contemporary treatment modalities, such as chemotherapy, targeted therapies, chimeric antigen receptor T-cell therapy, and hematopoietic stem cell transplantation, have contributed to improved patient outcomes (3). However, relapse and the development of drug resistance remain significant therapeutic challenges, leading to disease progression and poor prognosis (4). Therefore, there is an urgent need to identify novel prognostic biomarkers and molecular signatures that can facilitate risk stratification, guide treatment selection, and ultimately improve clinical outcomes for patients with MM.

Of particular interest in MM research is the intricate involvement of mitochondria, the cellular powerhouses and key regulators of PCD (5). Subtle alterations in mitochondrial function and regulatory mechanisms profoundly influence the cell’s fate (6, 7). In cancer cells such as those found in MM, mitochondrial bioenergetics are often reprogrammed to support the heightened energetic demands associated with rapid proliferation and metastasis (8). Mitochondrial functional and structural abnormalities are pivotal drivers of tumor formation and progression, wherein mitochondria facilitate the malignant transformation of tumor precursor cells through the production of reactive oxygen species, aberrant accumulation of specific metabolites, and functional defects (9, 10). Furthermore, the rapid advancements in molecular biology and genomics have unveiled the intricate biological characteristics of MM, shedding light on the dysregulation of intracellular and intercellular signaling pathways that are crucial for the proliferation and survival of cancer cells (11, 12). Within this framework, PCD serves as an essential homeostatic mechanism (13). The diversity and complexity of PCD occupy a pivotal position in oncology (14, 15). PCD manifests in diverse forms, including apoptosis, necrosis, and autophagy, each playing a critical role in eliminating damaged or abnormal cells, regulating cell populations, and shaping tissue development and homeostasis (16). The intricate interplay between mitochondrial dynamics and cell death pathways, particularly their regulation of plasma cell survival and response to therapy, has become a central focus of research (17–19). Mitochondrial membrane potential changes (20), cytochrome c release (21, 22), Bcl-2 protein family interactions (23), and other molecular events form a complex network that influences MM cell survival, pathogenesis, and treatment responsiveness (24). This underscores the critical importance of further exploring the interconnected roles of mitochondria and PCD in the pathophysiology and treatment of MM.

The central objective of our study is to comprehensively investigate the functional roles, expression profiles, and clinical correlations of MRGs and PCDRGs in MM. To achieve this, we have integrated bioinformatics approaches with large-scale transcriptomic data analysis. Our specific aims include the identification of critical prognostic markers among MRGs and PCDRGs, and the subsequent construction of a risk scoring model to predict patient outcomes accurately. Through an in-depth analysis of the immune microenvironment, we aim to disentangle the complex immune interactions related to MM and elucidate their role in disease progression and therapeutic response.

Furthermore, we have explored the locations and interactions of MRGs and PCDRGs within the regulatory network, providing theoretical support for the development of immunotherapeutic strategies targeting these pathways. Additionally, drug sensitivity analysis has guided the selection of potential inhibitory candidates for MM cells, paving the way for therapeutic optimization and the development of novel targeted therapies. Ultimately, our research endeavors to enhance the fundamental understanding of the biological characteristics of MM, offer more precise biomarkers and therapeutic targets for the treatment and prognostic evaluation of MM, and ultimately improve clinical outcomes for patients afflicted with this debilitating disease.

## 2 Materials and methods

### 2.1 Data collection

859 samples were obtained from UCSC Xena’s MMRF-COMPASS database, including 764 primary MM samples (763 with survival data). We processed the count, FPKM, survival, and phenotypic data to obtain expression matrices (genes as rows, samples as columns) and tables with grouping, survival, and phenotypic information for prognostic modeling. See **Supplementary Table 1** for baseline details.

MM-related datasets GSE47552 (99 samples: 5 controls, 20 MGUS, 33 SMM, 41 MM), GSE4581 (414 MM), GSE24080 (559 MM), and GSE6477 (134 samples: 15 controls, 21 MGUS, 23 SMM, 75 MM) were downloaded from GEO. We converted probe IDs to gene names and constructed expression matrices and grouping information for differential expression and prognostic model validation.

1136 MRGs were obtained from MitoCarta3.0, and 1548 PCDRGs from the literature.

### 2.2 Differential expression analysis

Differential expression analysis was performed using the limma package (version 3.52.4) (25) to identify differentially expressed genes (DEGs) (adj. *p* < 0.05 and |log2FoldChange (FC)| > 0.5). Volcano map of DEGs were drafted by ggplot2 (version 3.3.6) (26). Top 20 up- and down-regulated DEGs in |log2FC| sequencing were displayed using Complex Heatmap package (version 2.12.1) (27).

### 2.3 Identification and enrichment analysis of candidate genes

Candidate genes were obtained by taking intersection of DEGs, MRGs and PCDRGs, and results were visualized by ggvenn package (version 0.1.9) (28). Expression of candidate genes was visualized by heatmap. Subsequently, the candidate genes were analyzed for GO and KEGG enrichment (*p* < 0.05) using the cluster analysis software package (version 4.7.1.001). Top8 GO terms and KEGG pathways of p value were visualized by enrichplot package (version 1.8.1) (29). Next, ssGSEA algorithm was used to calculate ssGSEA score of MM patients by GSVA package (version 1.50.1) (30) in MMRF-COMMPASS dataset. Then MM samples were divided into high and low scoring groups based on median value of ssGSEA score. Survival difference of 2 scoring groups was compared by log-rank test.

### 2.4 Screening of prognosis genes

Univariate Cox regression analysis was performed in MMRF-COMMPASS dataset by survival package (version 3.4-0) (31) to find survival-related genes (HR ≠ 1, *p* < 0.05). Then, genes that passed proportional hazard (PH) assumption test were then used for subsequent analysis (*p* > 0.05). After that, LASSO regression analysis was used to further screen candidate prognosis genes by glmnet package (version 4.1-6) (32). Furthermore, multivariate Cox and stepwise regression analyses were performed to screen prognostic genes and calculate the expression of MM samples. KM survival curve of prognosis genes was utilized to compare survival difference between 2 scoring groups using log-rank test. Subsequently, consensus clustering of MM samples was processed by Consensus Cluster Plus package (version 3.18) (33) relied on expression mode of prognosis genes. MM samples were divided into different subtypes, and KM survival analysis of different subtypes was proceeded.

### 2.5 Construction of risk model

In order to further evaluate the efficacy of the risk model in predicting patient survival and treatment response, time-dependent ROC curves were drafted by time ROC package (version 0.4) (34). The MM patients were stratified into high and low risk group, and survival differences between the 2 risk groups were compared by KM survival curves. Additionally, the performance of the risk model was validated in the GSE4581 and GSE24080 datasets. Furthermore, to explore the relationship between risk score and clinical features, correlation analysis was conducted using three clinical features (age, gender, and ISS stage) in the two risk cohorts. The proportions of the 3 clinical characteristics Age, Gender and ISS Stage in the high and low risk groups are shown in the graph with different percentages and color divisions. By knowing the percentage shares, we can assess their potential impact on prognosis and also guide the development of personalized treatment strategies. Then differences in risk scores across different clinical features were compared by Wilcoxon test (*p* < 0.05).

### 2.6 Construction of nomogram

Univariate Cox, PH assumption test, and multivariate Cox regression analyses were processed to find independent prognostic factors among risk score, age, gender and ISS stage. After obtaining, a nomogram was constructed to predict survival probability of MM. Predictive ability of nomogram was evaluated by calibration curve, ROC and decision curve analysis (DCA).

### 2.7 Gene Set Enrichment Analysis and chromosome localization

To explore corresponding functions and pathways involved in risk groups, differential analysis between 2 risk groups was performed by DESeq2 package in MMRF-COMMPASS dataset (version 1.36.0) (35). Based on background genes which were downloaded from MSigDB database, GSEA was processed by clusterProfiler package. Chromosome localization of prognosis genes was determined through RCircos package (version 1.2.2) (36).

### 2.8 Immune microenvironment analysis

Difference of immune cells between 2 risk groups was compared. Firstly, ssGSEA algorithm was used to calculate score of 28 immune cell types in all samples of MMRF-COMMPASS dataset by GSVA package. Then difference of immune cells of 2 risk groups was compared by wilcoxon test (*p* < 0.05). In addition, 12 gene sets of immune related functions were obtained from published literature (37). Immune-related function scores were calculated using the ssGSEA algorithm in the GSVA software package and compared between the two risk groups (*p* < 0.05). Paradoxical correlation analysis was also performed to explore the relationship between prognostic genes and risk scores with different immune cells and immune-related functions. Correlation results were presented using the ggcor and ggplot2 software packages, respectively.

### 2.9 Correlation analysis of immunotherapy

The effect of immune checkpoints was explored by analyzing the differential expression of 38 immune checkpoints (38) in MM samples from both groups. The relationship between risk scores and differences in immune checkpoints was investigated using Spearman’s correlation analysis (*p* < 0.05). TIDE, MDSC, CAF, TAM, merck18, dysfunction and exclusion scores were compared between the two risk groups (*p* < 0.05) and their correlation with risk scores was analyzed. In addition, differences in immunity scores, stromal scores and estimated scores were investigated. Somatic mutations in multiple myeloma patients in the MMRF-COMPASS dataset were analyzed using the maftools software package (version 2.12.0) (39), and the mutation frequencies of the top 20 genes were shown by waterfall plots.

### 2.10 Regulatory mechanism and drug analysis

To explore miRNAs targeting prognosis genes. The intersection of miRNAs extracted from both starBase and miRTarBase databases were identified as key miRNAs. Then long non-coding RNAs (lncRNAs) targeting key miRNAs were obtained from miRNet and starBase (clipExpNum > 5) databases. After that, key lncRNAs were selected by overlapping lncRNAs. Finally, key miRNAs, key lncRNAs and prognosis genes with interactions were constructed into a lncRNAs-miRNAs-mRNAs regulatory network.

Half maximal inhibitory concentration (IC50) value of 198 drugs which were obtained from GDSC was calculated by oncoPredict package (version 0.2) (40). Difference of IC50 value of 198 drugs of different risk groups was compared (*p* < 0.05). Expression of top 6 drugs with p value of Wilcoxon test was displayed by boxplot. To further explore correlation of risk score and IC50 value, spearman correlation analysis was performed (*p* < 0.001).

Furthermore, potential drugs of MM were predicted by CTD relied on prognosis genes (Reference Count ≥ 2). Drug-prognosis genes network was visualized by cytoscape. Protein structures of prognosis genes were obtained from PDB, and SDF format files of therapeutic drugs were sourced from the PubChem database. Preparation and preprocessing of drug and gene protein structures were performed using AutoDock tool (version 1.1.2) (41).

### 2.11 Expression validation of prognosis genes

The expression trends of prognosis genes in the MM and control groups in the GSE47552 and GSE6477 datasets were analyzed by wilcoxon test. Next, the expression of prognosis genes in different subtypes was compared by kruskal test.

### 2.12 RNA extraction and quantitative real-time PCR

For quantification of gene expression profiles, we performed qRT-PCR on mononuclear cells from samples of multiple myeloma patients bone marrow before treatment (MM, = 36) and healthy controls (=24) from the Second Affiliated Hospital of Xi’an Jiaotong University. Sample total RNA was extracted using TRIzol reagent (Thermo Fisher Scientific, USA), cDNA synthesis was accomplished with PrimeScript ™ RT Master Mix (Takara Bio, USA), and then amplification reactions were carried out in a real-time PCR system using TB Green^®^ Premix Ex Taq (Takara Bio, USA). Each sample was evaluated three times to ensure accuracy. GAPDH was used as a housekeeping gene for normalization, and CT values were recorded for relevant and reference genes in each sample. Relative expression levels of target genes were calculated using the 2^^-ΔΔCT^ method. The Wilcoxon rank sum test was used to compare gene expression between the MM and control groups. Detailed qRT-PCR primer sequences are shown in **Supplementary Table 2**.

### 2.13 TISCH analysis

We used the TISCH repository (Tumor Immune Single-cell Hub, available at http://tisch.comp-genomics.org/) to assess the presence of potential tumour antigens in immune cells penetrating the bone marrow. The GSE1151310 dataset in this repository is divided into four main cell categories, visualizing the expression patterns of specific genes in different types of immune cells.

### 2.14 Statistical analysis

R software (version 4.3.2) was used to process and analyze data. All statistical tests were two-sided. The *p* value < 0.05 was considered statistically significant. Differences in parameters between high and low risk groups were tested using the wilcox.test method.

## 3 Results

### 3.1 Identification and analysis of 30 candidate genes

**Figure 1:**
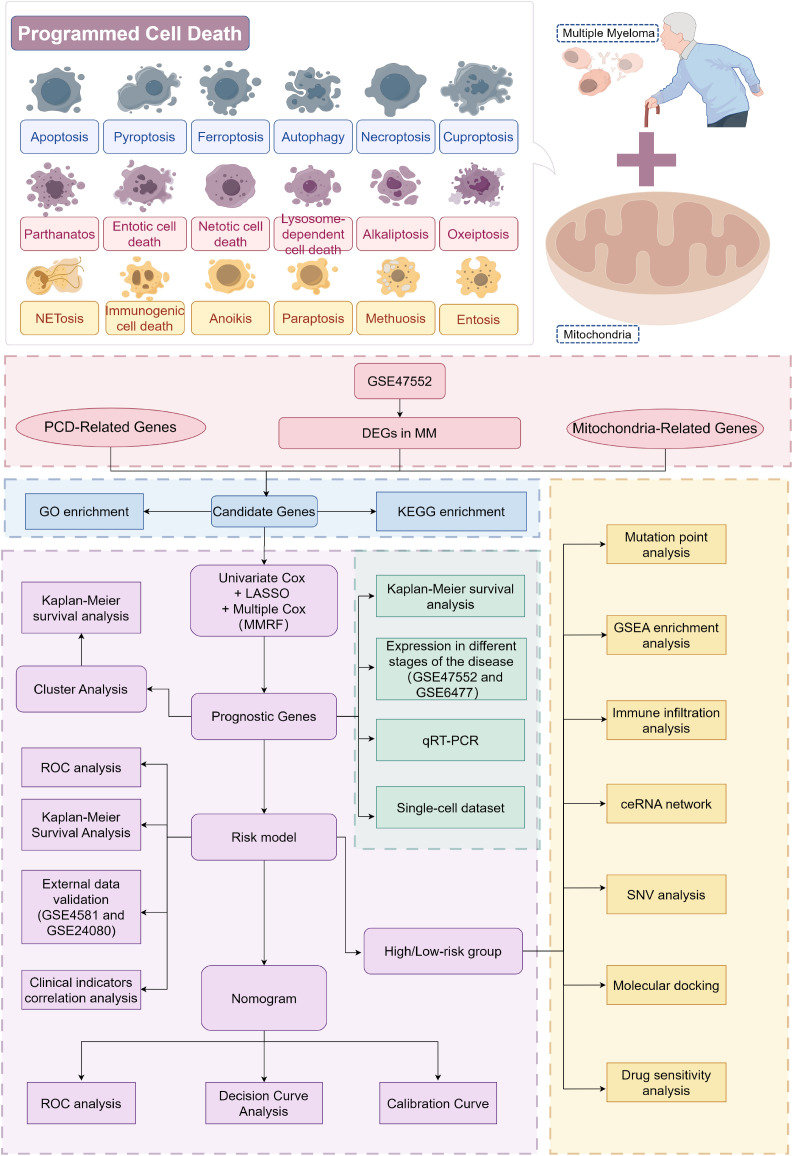
Flowchart for studying mitochondrial function and programmed cell death patterns in multiple myeloma. From the GSE47552 dataset, DEGs between MM and control samples were screened and intersected with MRGs and PCDRGs to obtain candidates (The pink part in the figure). GO and KEGG enrichment analyses were performed on these candidates (The blue part in the figure). Prognostic genes were then identified through univariate and multivariate Cox regression, with Kaplan-Meier curves plotted and cluster analyses conducted. A risk model was constructed based on the prognostic genes and validated in two independent sets. Independent prognostic factors were screened, and a nomogram was built to evaluate predictive ability (The purple part in the figure). Expression differences of the prognostic genes between MM and controls were validated (The green part in the figure). Finally, GSEA enrichment and immune infiltration analyses were performed comparing high- and low-risk groups (The yellow part in the figure). This study elucidates molecular mechanisms underlying MM development and potential therapeutic targets by examining 18 cell death forms and mitochondrial roles in MM.

**Figure 2:**
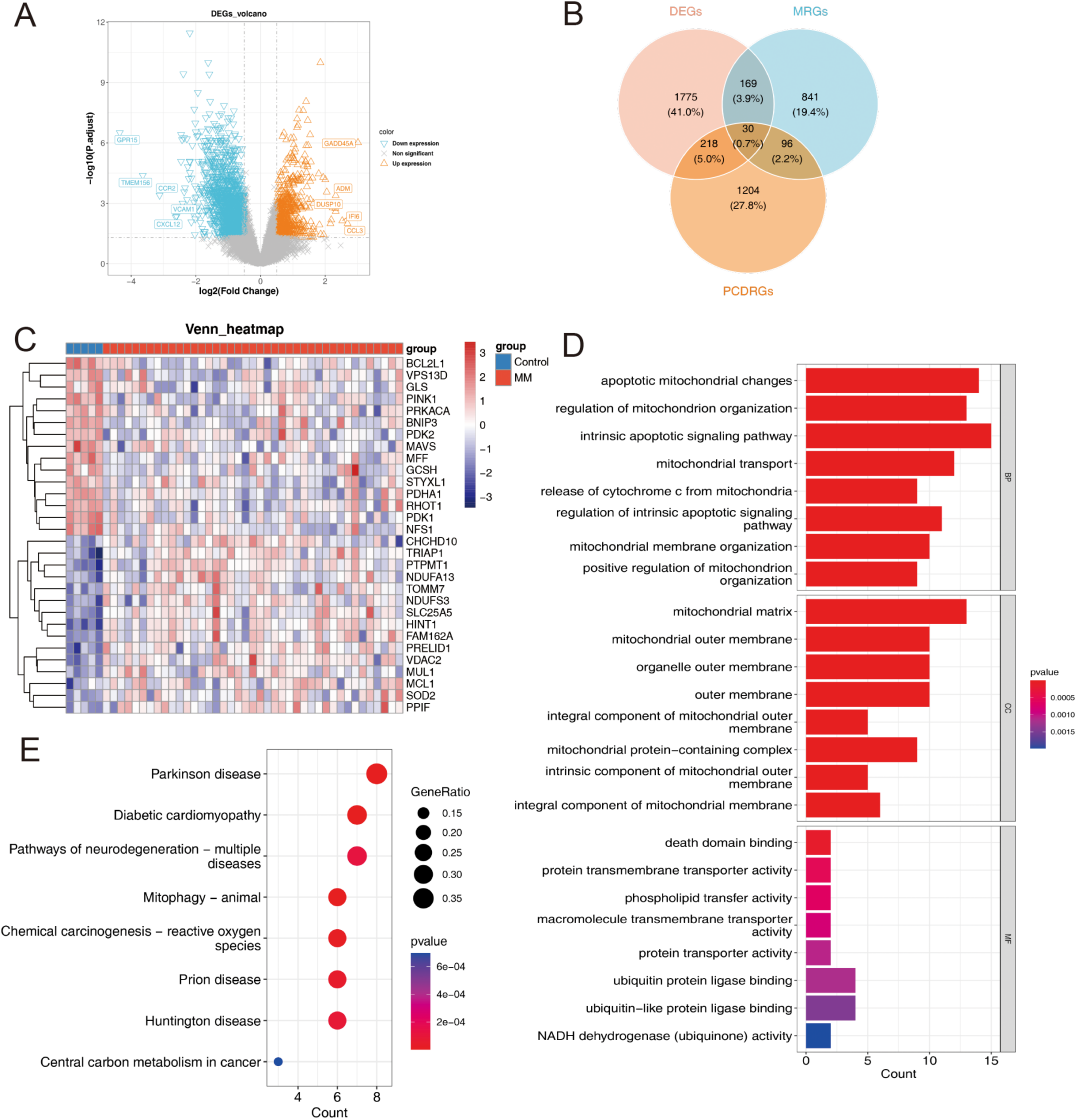
**(A)** Volcano plot of DEGs in the GSE47552 dataset. **(B)** A Venn diagram showing the intersection of DEGs, MRG and PCDRGs. **(C)** Candidate genes expression heatmap. **(D)** GO Analysis for candidate genes: Categorized by Biological Process (BP), Cellular Component (CC) and Molecular Function (MF). **(E)** Kyoto Encyclopedia of Genes and Genomes Pathways for candidate genes.

**Figure 1** illustrates the study’s flowchart (By Figdraw). A comprehensive differential expression analysis identified a total of 2,192 DEGs, comprising 595 upregulated and 1,597 downregulated DEGs, in MM samples compared to control samples (**Figure 2A**). The heatmap of DEGs in MM and control groups is presented in **Supplementary Figure 1**. Thirty candidate genes were obtained by intersecting the 2,192 DEGs with 1,136 MRGs and 1,548 PCDRGs (**Figure 2B**). The heatmap showcased the high and low expression patterns of these candidate genes across the samples (**Figure 2C**).

GO and KEGG enrichment analyses revealed that the candidate genes were significantly enriched (*p* < 0.05) in 574 GO terms and 33 KEGG pathways. In the GO analysis, the candidate genes were enriched in intrinsic apoptotic signaling pathways, apoptotic mitochondrial changes, regulation of mitochondrion organization were enriched by candidate genes (**Figure 2D**). Regarding KEGG pathways, the candidate genes were primarily enriched in lipoic acid metabolism, apoptosis, central carbon metabolism in cancer, and associated pathways (**Figure 2E**). These findings indicate a strong correlation between the candidate genes and mitochondrial function, programmed cell death, and cancer development.

Furthermore, MM samples were stratified into high and low scoring groups based on the median value of the ssGSEA score calculated from the candidate genes. Survival analysis demonstrated that patients in the low scoring group exhibited poorer overall survival compared to those in the high scoring group (**Supplementary Figure 2**).

### 3.2 TRIAP1, TOMM7, PINK1, CHCHD10, PPIF, BCL2L1 and NDUFA13 were identified as prognosis genes

**Figure 3:**
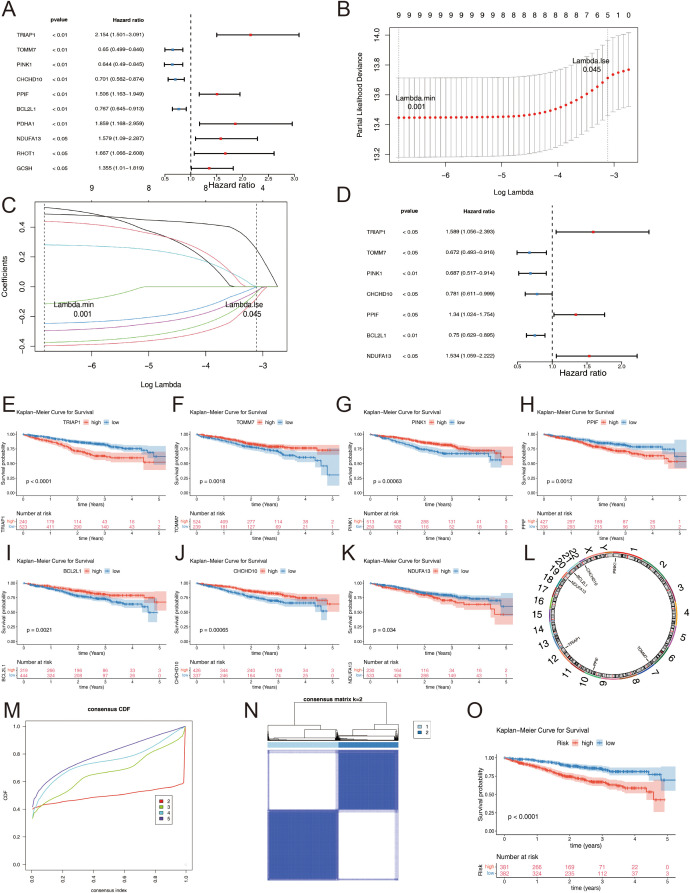
**(A)** Univariate Cox regression analysis of candidate genes. **(B)** Sample clustering consistency assessing the coherence of group assignments. **(C)** LASSO regression selected the best predictive variables through 10-fold cross-validation. **(D)** Multivariate Cox demonstrated that 7 genes were protection factors. **(E–K)** Prognostic genes KM curve: **(E)** TRIAP1; **(F)** TOMM7; **(G)** PINK1; **(H)** PPIF; **(I)** BCL2L1; **(J)** CHCHD10; **(K)** NDUFA13. **(L)** Chromosomal localization of prognostic genes. **(M–O)** Consensus Clustering Analysis: **(M)** Cumulative distribution curve depicting the distribution of values. **(N)** Cluster heatmap visualizing patterns across subgroups. **(O)** KM survival curves comparing survival outcomes among different MM subgroups.

Following the identification of candidate genes, univariate Cox regression analysis was performed to screen for survival-related genes, resulting in the selection of 10 genes (**Figure 3A**). Nine out of these 10 survival-related genes (*TRIAP1, TOMM7, PINK1, CHCHD10, PPIF, BCL2L1, PDHA1, NDUFA13*, and *GCSH*) passed the proportional hazards (PH) assumption test (*p* > 0.05) and were subsequently included in the analysis. Further, the LASSO regression analysis identified all 9 genes as candidate prognostic genes (Lambda min = 0.001) (**Figures 3B, C**).

Multivariate Cox regression and stepwise regression analyses were then performed, revealing that *TRIAP1, TOMM7, PINK1, CHCHD10, PPIF, BCL2L1*, and *NDUFA13* were significant prognostic genes. Results from the multivariate Cox regression demonstrated that *TOMM7, PINK1, CHCHD10*, and *BCL2L1* were protective factors, whereas *TRIAP1, PPIF*, and *NDUFA13* were risk factors (**Figure 3D**). KM survival curves showed that patients with high expression levels of *TOMM7, PINK1, CHCHD10*, and *BCL2L1* had poorer survival outcomes, while those with high expression levels of *TRIAP1, PPIF*, and *NDUFA13* exhibited better survival (**Figures 3E–K**). Chromosome localization analysis revealed that *PINK1, TOMM7, PPIF, TRIAP1, NDUFA13, BCL2L1*, and *CHCHD10* are located on chromosomes 1, 7, 10, 12, 19, 20, and 22, respectively (**Figure 3L**).

Based on the expression patterns of the prognostic genes, consensus clustering was performed, which stratified the MM samples into two subtypes (cluster 1: cluster 2 = 414: 349) (**Figures 3M, N**). To evaluate the reliability of consensus clustering, a KM survival curve was plotted, and survival difference analysis demonstrated that samples in cluster 1 had a better overall survival compared to those in cluster 2 (*p* < 0.0001) (**Figure 3O**).

### 3.3 A risk model was constructed to predict the survival probability of MM patients

A risk model was constructed based on the identified prognostic genes, and the risk scoring formula was derived as follows: risk score = (0.5887) × expression level of *TRIAP1* + (-0.5210) × expression level of *TOMM7* + (-0.3938) × expression level of *PINK1* + (-0.1795) × expression level of *CHCHD10* + (-0.2508) × expression level of *PPIF* + (-0.2562) × expression level of *BCL2L1* + (0.4431) × expression level of *NDUFA13*.

**Figure 4:**
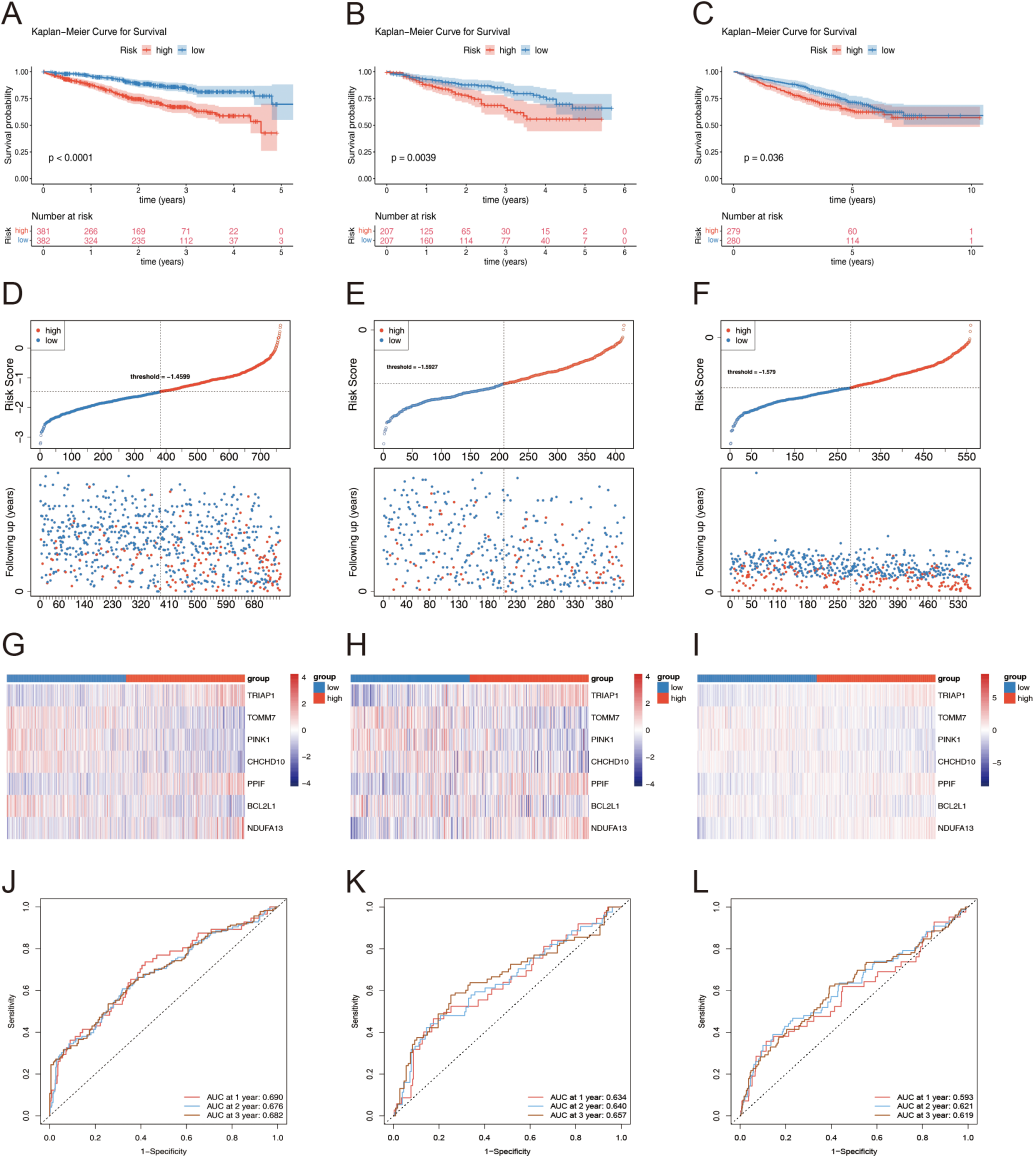
**(A–C)** KM Survival Curves Highlighting Differences Between Risk Groups: **(A)** MMRF. **(B)** GSE4581. **(C)** GSE24080. **(D–F)** Risk factor plot for the high-risk and low-risk groups: **(D)** MMRF. **(E)** GSE4581. **(F)** GSE24080. **(G–I)** Heatmap of prognostic gene expression levels in high and low-risk groups: **(G)** MMRF. **(H)** GSE4581. **(I)** GSE24080. **(J–L)** Time-Dependent ROC Curves Assessing the Accuracy of Survival Predictions: **(J)** MMRF. **(K)** GSE4581. **(L)** GSE24080.

We validated this risk model across three distinct datasets by evaluating patient survival stratified by their calculated risk scores. KM curves demonstrated a distinct separation between high- and low-risk groups within the MMRF training dataset, exhibiting significant differences in overall survival (*p* < 0.0001) (**Figure 4A**). This stratification was similarly observed in the validation datasets GSE4581 (**Figure 4B**) and GSE24080 (**Figure 4C**), both exhibiting statistically significant separations (*p* < 0.05). After stratifying patients according to ascending risk scores, we generated risk curve plots for each dataset (**Figures 4D–F**). These graphs revealed a higher mortality rate in the high-risk group compared to the low-risk group, indicating a lower mortality rate and improved survival outcomes for patients classified as low-risk in MM. The heatmap visualizing the differential expression of prognostic genes across datasets (**Figures 4G–I**) demonstrated downregulation of *TOMM7, PINK1, CHCHD10*, and *BCL2L1* in the high-risk group across all datasets (MMRF in **Figure 4G**, GSE4581 in **Figure 4H**, and GSE24080 in **Figure 4I**). Conversely, *TRIAP1, PPIF*, and *NDUFA13* were upregulated in the same group, reinforcing their roles as potential risk factors.

Time-dependent ROC curve analyses further substantiated the robustness of our risk score across datasets. In the MMRF dataset, the area under the ROC curve (AUC) at 1, 2, and 3 years was 0.690, 0.676, and 0.682, respectively (**Figure 4J**). For GSE4581, AUC values were 0.634, 0.640, and 0.657 (**Figure 4K**), while GSE24080 exhibited AUCs of 0.5593, 0.621, and 0.619 at the same time points (**Figure 4L**). Collectively, these results underscore the consistent performance and reliability of our risk prediction model across various datasets.

**Figure 5:**
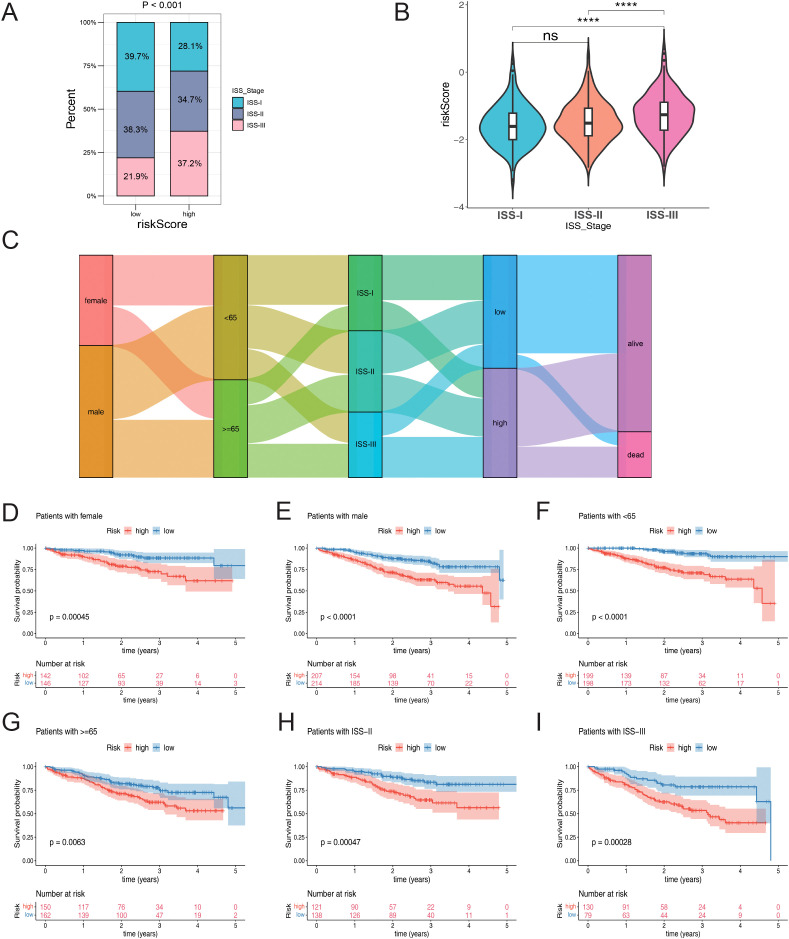
**(A)** The proportion of ISS staging clinical feature subgroups in high and low-risk groups. **(B)** Risk score differences among the clinical feature subgroups of the ISS staging system. **(C)** Sankey diagram associated with different clinical feature subgroups. **(D–I)** KM curves for high and low-risk groups within different clinical feature subgroups: **(D)** Female; **(E)** Male; **(F)** Age < 65; **(G)** Age >= 65; **(H)** ISS-II; **(I)** ISS-III. Statistical significance is indicated as follows: ****p < 0.0001, ns = no statistical significance.

Moreover, correlation analysis of risk score and clinical features showed ISS stage had significant difference between 2 risk groups (*p* < 0.0001), and ISS-III had higher proportion in higher risk group (**Figure 5A**). Besides, risk score was also significantly differential in ISS stage. From ISS-I to ISS-III, corresponding risk score was higher and higher in turn (**Figure 5B**). The Sankey diagram effectively distinguished patients with ISS Stage II into high and low risk groups. Notably, a considerable proportion of patients with ISS Stage III fell into the high-risk category, while the majority of patients in the low-risk group survived (**Figure 5C**). In the clinical subgroup analysis, significant differences in clinical features were observed between the two risk groups, irrespective of the patient’s gender, age (whether over 65 years or not), and ISS Stage (II or III) (**Figures 5D–I**).

### 3.4 A nomogram was established to predict the survival of MM

**Figure 6:**
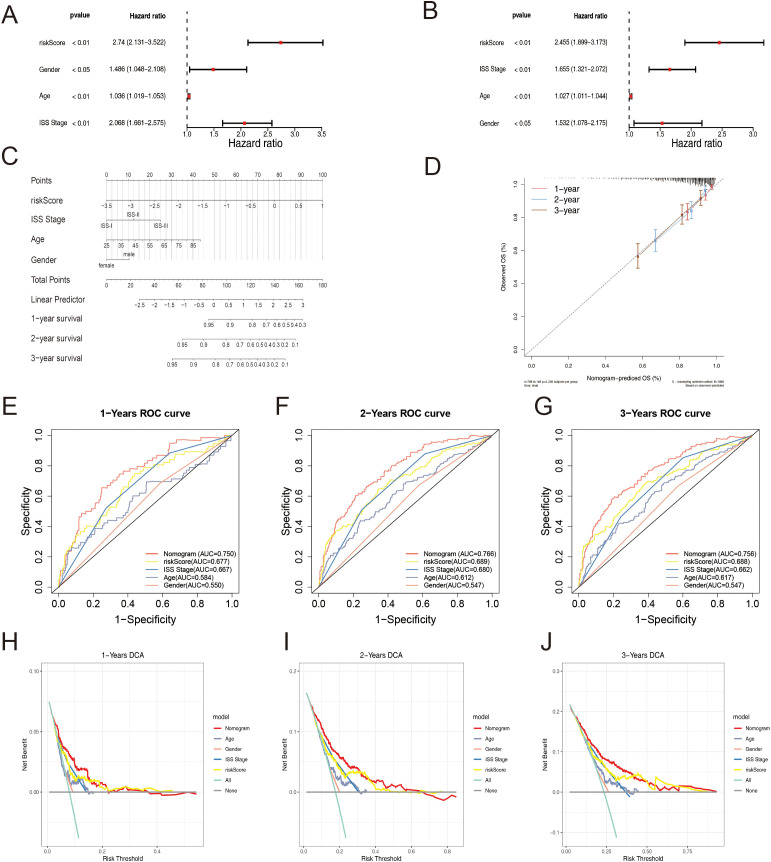
**(A)** Univariate Cox regression analysis depicting the association between clinical parameters and the risk score. **(B)** Multivariate Cox regression analysis elucidating the combined effects of clinical parameters and the risk score. **(C)** Nomogram presenting the scores assigned to each factor and the aggregated prognostic indicator. **(D)** Calibration Curves for the Prognostic Model at Different Time. **(E–G)** ROC Curves for Overall Survival Over Time Based on Various Factors and the Nomogram: **(E)** 1 Year; **(F)** 3 Years; **(G)** 5 Years. **(H–J)** Decision Curve Analysis for Different Factors and the Nomogram at Various Time: **(H)** 1 Year; **(I)** 3 Years; **(J)** 5 Years.

To evaluate the prognostic utility of the identified gene signature in conjunction with clinical parameters, a comprehensive analysis was conducted. Initially, univariate Cox regression analysis and assessment of proportional hazards (PH) assumption were performed to identify potential independent prognostic factors. Subsequently, multivariate Cox regression analysis confirmed the independent prognostic significance of the risk score, age, gender, and International Staging System (ISS) stage for MM patients (**Figures 6A, B**). All these factors were identified as independent risk factors for MM.

Based on the identified independent prognostic factors, a nomogram was constructed to facilitate the prediction of overall survival rates in MM patients (**Figure 6C**). The calibration curve demonstrated an excellent concordance between the predicted and observed survival probabilities (**Figure 6D**), the predicted lines closely aligned with the diagonal, indicating a good fit and our model’s ability to make accurate predictions. The AUC values exceeded 0.7 across multiple time points, indicating satisfactory discriminatory ability (**Figures 6E–G**). These results collectively underscore the robust predictive performance of the nomogram.

Furthermore, DCA revealed a clinically significant net benefit associated with the nomogram, surpassing the predictive utility of individual factors, including the ISS stage (**Figures 6H–J**). The ROC and DCA analyses of the prediction model exhibited superior performance compared to the ISS stage alone. These findings highlight the potential clinical utility of the nomogram in guiding therapeutic decisions and risk stratification for MM patients, offering improved prognostic stratification compared to the conventional ISS staging system.

### 3.5 Analysis of mutations and corresponding pathways

**Figure 7:**
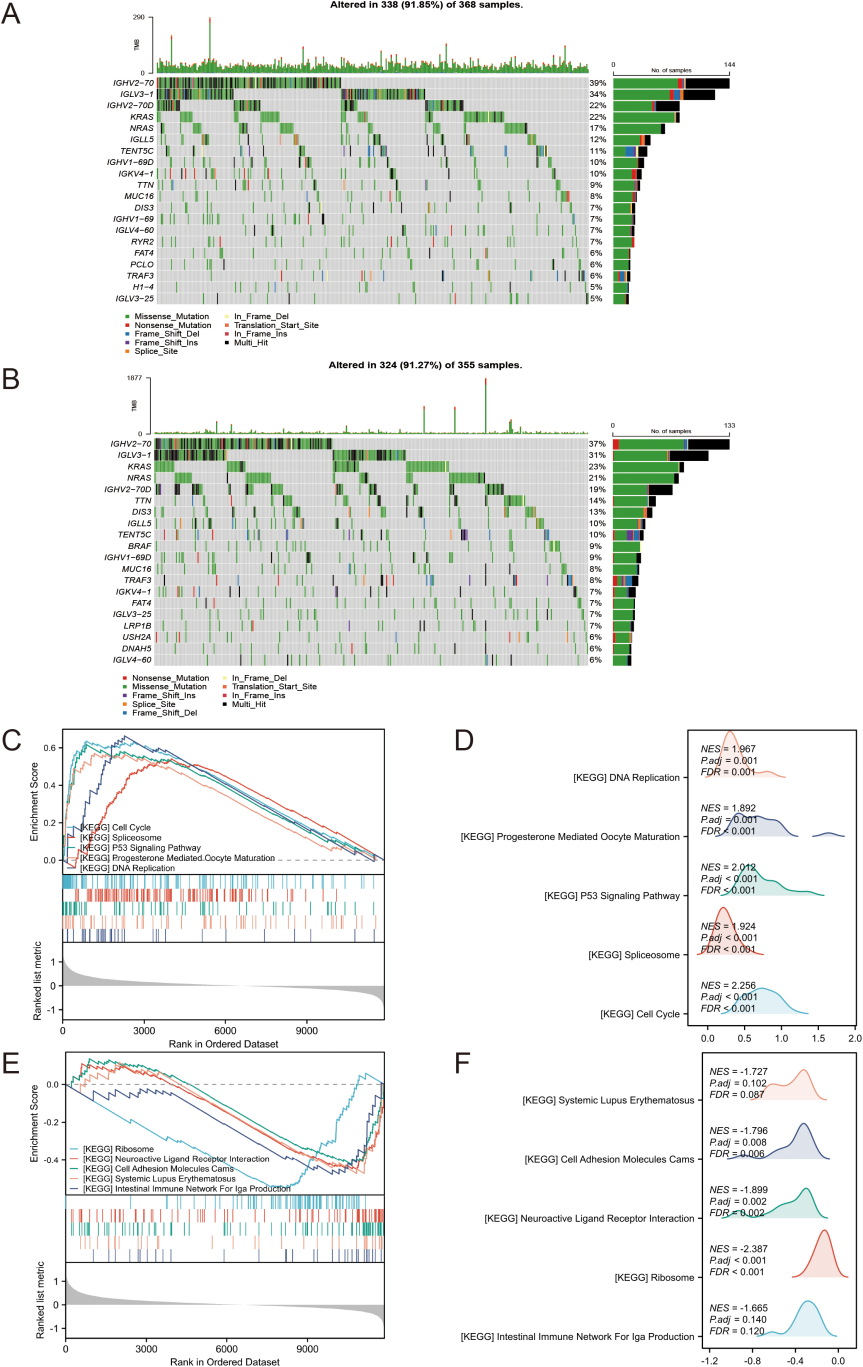
**(A)** Waterfall plot of the top 20 most frequently mutated genes in the low-risk group. **(B)** Waterfall plot of the top 20 most frequently mutated genes in the high-risk group. **(C–F)** GSEA of DEGs between high and low-risk groups. **(C, D)** Upregulated. **(E, F)** Downregulated.

To investigate the primary genetic mutations among different risk groups, we analyzed the somatic mutations in tumors from multiple myeloma patients in the MMRF-COMMPASS dataset. We constructed waterfall plots for the top 20 most frequently mutated genes in both the high-risk and low-risk groups. The plots revealed that the top three genes with the highest mutation frequencies in the low-risk group were IGHV2-70, IGLV3-1, and IGHV2-70D (**Figure 7A**). In the high-risk group, the top three genes with the highest mutation frequencies were IGHV2-70, IGLV3-1, and KRAS (**Figure 7B**). To further explore the corresponding signaling pathways and biological mechanisms involved in the two risk groups, we performed GSEA. The results showed that 28 pathways were significantly enriched, and the top 5 up- and down-regulated pathways are displayed in **Figures 7C–F** (|normalized enrichment score (NES)| > 1, *p* < 0.05). The up-regulated pathways included cell cycle, p53 signaling pathway, DNA replication, and others (**Figures 7C, D**), indicates increased cell proliferation, dysregulated cell cycle control, and potential genomic instability in the high-risk tumors. Conversely, ribosome, neuroactive ligand-receptor interaction, and cell adhesion molecules (CAMs) were among the main down-regulated pathways (**Figures 7E, F**). These distinct molecular patterns may contribute to the aggressive clinical behavior and poor prognosis observed in the high-risk group.

### 3.6 Different immune microenvironment between different groups

**Figure 8:**
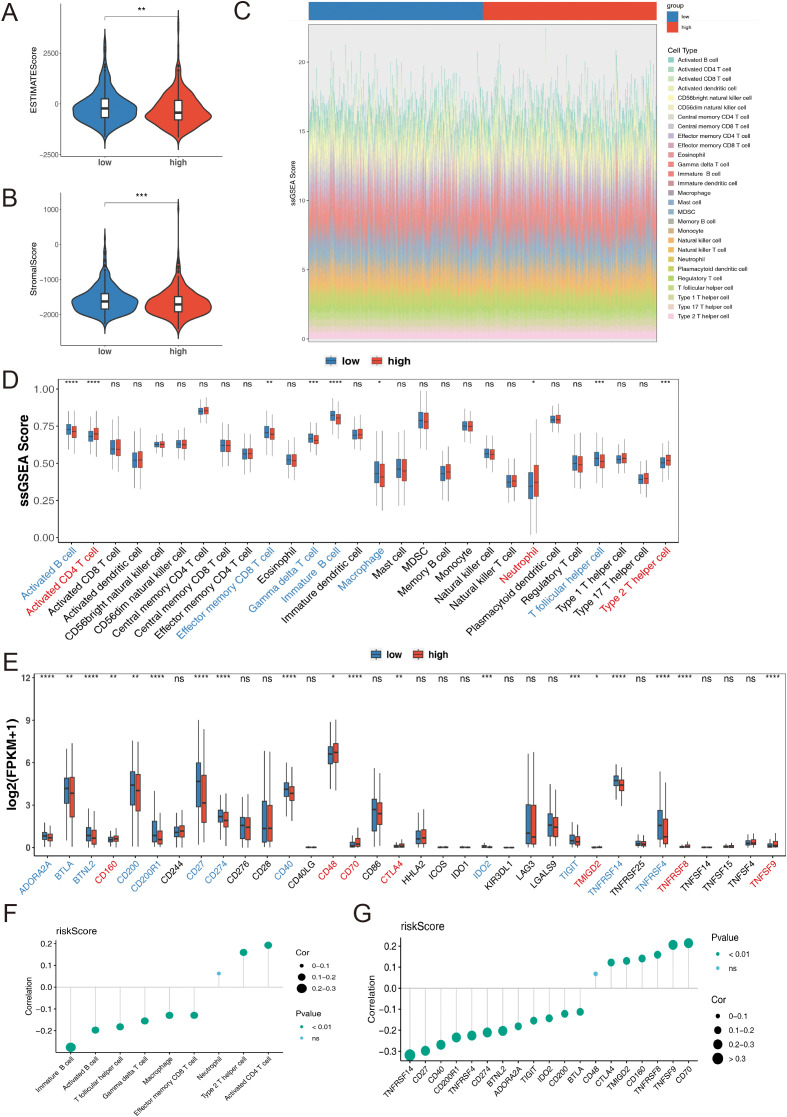
**(A)** ESTIMATE Score in high-and low-risk groups. **(B)** Stromal Score in high-and low-risk groups. **(C)** Stacked bar chart of immune cell infiltration proportions. **(D)** Immune Cells between high-and low-risk groups, utilizing the ssGSEA algorithm. **(E)** Immune Checkpoint between high-and low-risk groups. **(F)** Correlation between risk score and immune cells. **(G)** Correlation between risk score and immune checkpoints. Statistical significance is indicated as follows: *p < 0.05, **p < 0.01, ***p < 0.001, ****p < 0.0001, ns= no statistical significance.

We conducted a systematic and comprehensive analysis of the immune cell infiltration in both the high-risk and low-risk groups of MM patients. Utilizing the ESTIMATE algorithm, we found that patients in the low-risk group exhibited significantly higher ESTIMATE scores and Stromal scores compared to patients in the high-risk group (*p* < 0.01) (**Figures 8A, B**), indicating a more prominent presence of stromal and immune cells in the low-risk group. The infiltration levels of 28 immune cell types across the two risk groups are shown in **Figure 8C**. A total of 9 immune cell types, including effector memory CD8 T cells, gamma delta T cells, macrophages, neutrophils, and others, displayed remarkable differences between the two risk groups (**Figure 8D**). Additionally, a total of 19 immune checkpoints exhibited significant differences between the two risk groups. CD160, CD48, CD70, CTLA4, TMIGD2, TNFRSF8, and TNFSF9 were upregulated, while the remaining 12 immune checkpoints were downregulated in the high-risk group (**Figure 8E**). The risk score had a significantly positive correlation with activated CD4 T cells and a negative correlation with immune B cells (*p* < 0.05) (**Figure 8F**). Furthermore, the risk score exhibited the highest positive and negative correlations with CD70 and TNFRSF14, respectively (*p* < 0.01) (**Figure 8G**).

**Figure 9:**
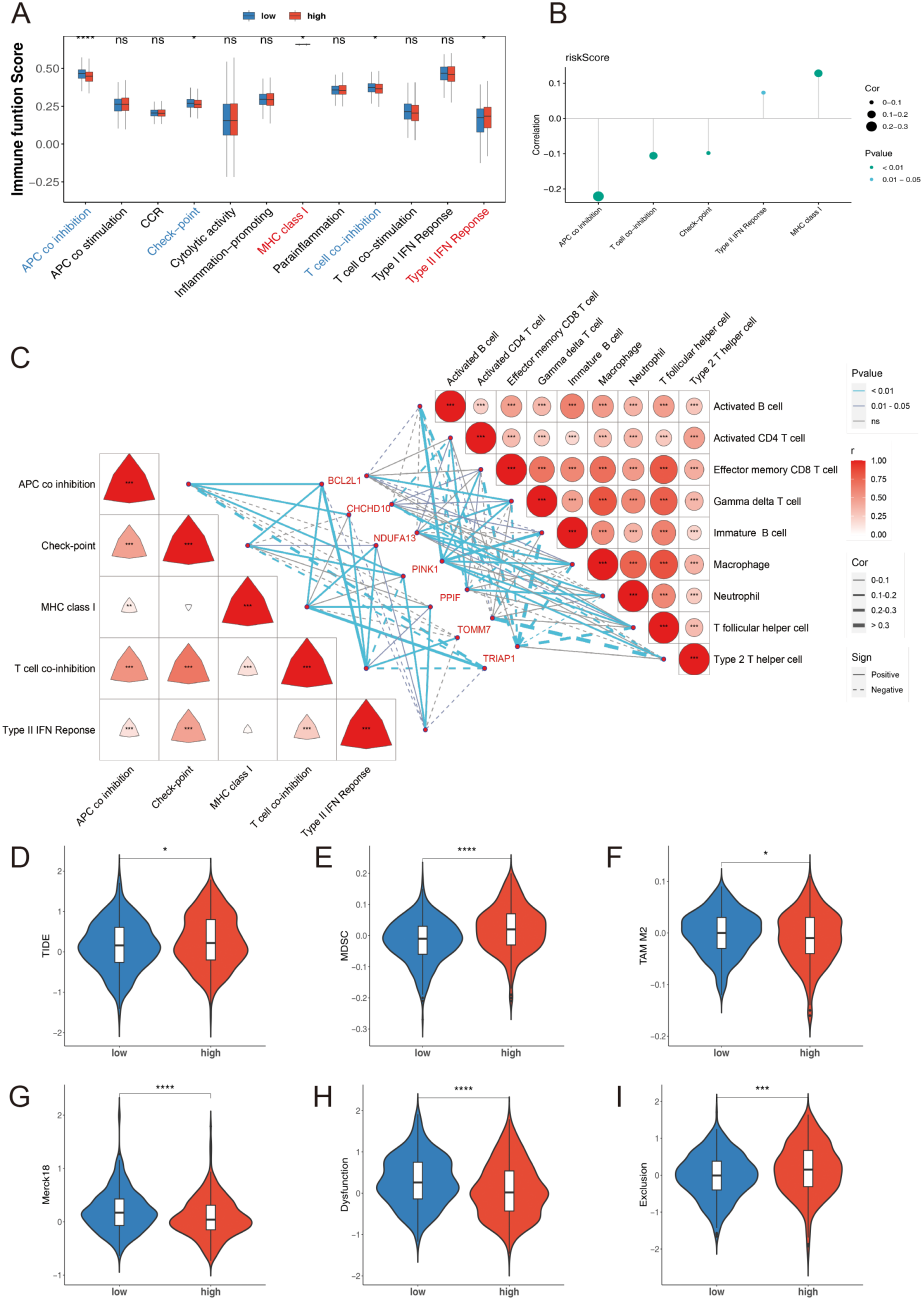
**(A)** Immune-related functions high-and low-risk groups, utilizing the ssGSEA algorithm. **(B)** Correlation between risk score and immune-related functions. **(C)** Heatmap of the correlation between prognostic genes and differential immune cells as well as immune-related functions. **(D)** TIDE Score. **(E)** MDSC Score. **(F)** TAM M2 Score. **(G)** Merck18 Score. **(H)** Dysfunction Score. **(I)** Exclusion Score. Statistical significance is indicated as follows: *p < 0.05, **p < 0.01, ***p < 0.001, ****p < 0.0001, ns= no statistical significance.

Additionally, five immune-related functions, including antigen-presenting cell (APC) co-inhibition, checkpoint, major histocompatibility complex (MHC) class I, T cell co-inhibition, and type II interferon (IFN) responses, exhibited significant differences between the two risk groups (**Figure 9A**). The risk score had a significantly positive correlation with MHC class I expression and a negative correlation with APC co-inhibition (*p* < 0.05) (**Figure 9B**). Results from the correlation analysis showed that PINK1 had the highest positive correlation with activated B cells, while TOMM7 had the highest negative correlation with type 2 T helper cells (*p* < 0.001). Regarding immune-related functions, BCL2L1 had the highest positive correlation with T cell co-inhibition, and TRIAP1 had the highest negative correlation with APC co-inhibition (*p* < 0.001) (**Figure 9C**).

To further investigate the possibility of tumor immune evasion, we utilized the Tumor Immune Dysfunction and Exclusion (TIDE) framework. The analysis revealed that the high-risk group had significantly higher TIDE scores (**Figure 9D**), myeloid-derived suppressor cell (MDSC) levels (**Figure 9E**), tumor-associated macrophage (TAM) M2 levels (**Figure 9F**), and Exclusion scores (**Figure 9I**) compared to the low-risk group (*p* < 0.05), indicating a higher likelihood of tumor immune evasion and a more immunosuppressive microenvironment in the high-risk group. Conversely, the high-risk group showed significantly lower levels of Merck18 scores (**Figure 9G**) and Dysfunction scores (**Figure 9H**) compared to the low-risk group (*p* < 0.001).

### 3.7 Regulatory networks and potential drugs of prognosis genes

**Figure 10:**
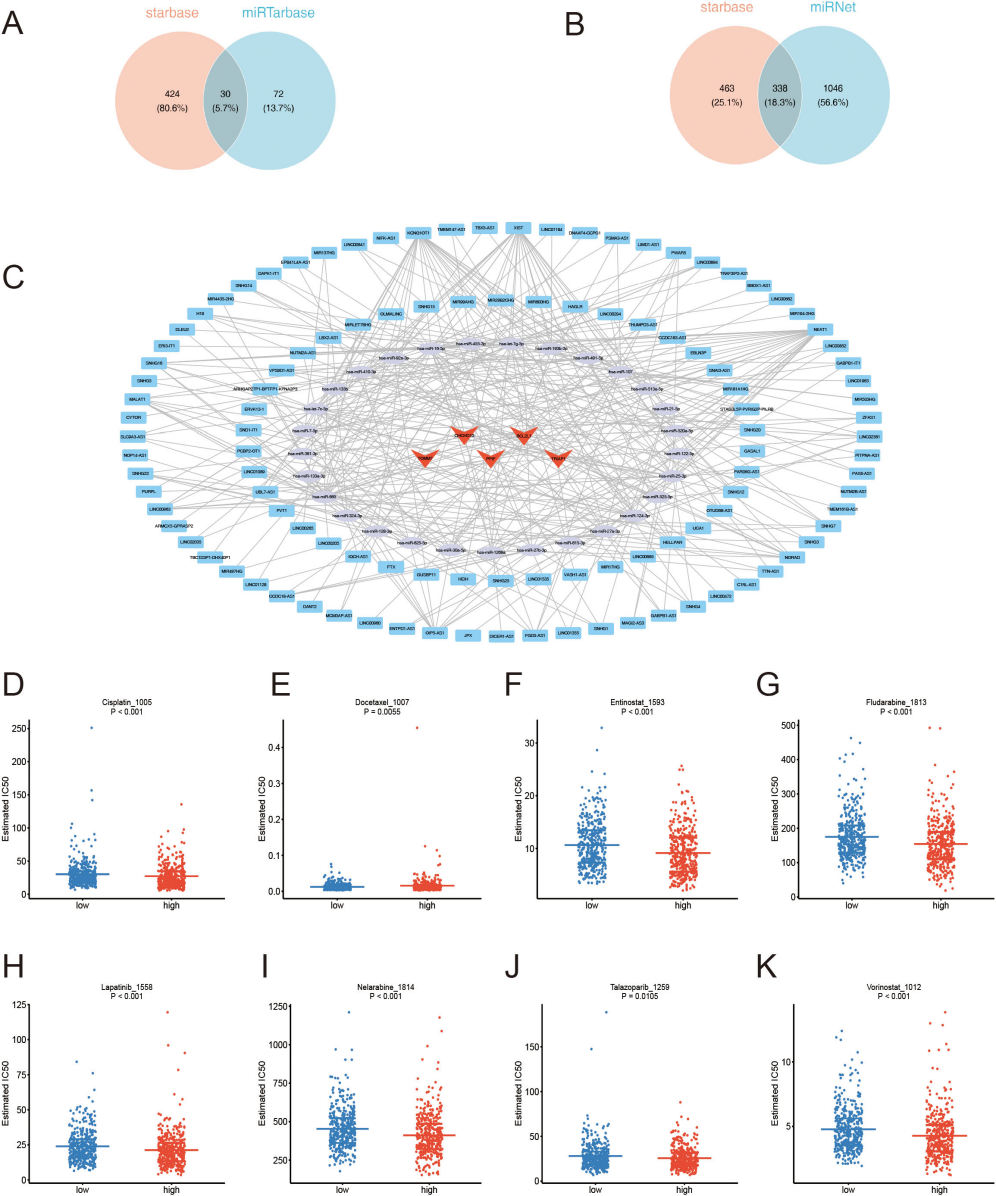
**(A)** miRNA Venn diagram. **(B)** lncRNA Venn diagram. **(C)** ceRNA network: Red triangles represent mRNAs, green circles represent miRNAs, and blue rectangles represent lncRNAs. **(D–K)** Estimated IC50 values comparing the drug sensitivity of high-and low-risk groups.

Based on the identified prognostic genes, 273 and 99 microRNAs (miRNAs) were respectively obtained from the starBase and miRTarBase databases. By taking the intersection of these miRNAs, 29 key miRNAs and 30 relational pairs were identified (**Figure 10A**). Next, 111 key long non-coding RNAs (lncRNAs) and 338 relational pairs were obtained by overlapping 516 and 340 lncRNAs, which were respectively predicted by the miRNet and starBase databases (**Figure 10B**). Finally, a lncRNA-miRNA-mRNA regulatory network was constructed comprising 5 mRNAs, 29 miRNAs, and 111 lncRNAs (**Figure 10C**). SNHG12, SNHG4, HEIH, and others are involved in the regulation of BCL2L1 via hsa-let-7c-5p. Similarly, MIR181A1HG, PAX8-AS1, TTN-AS1, and others regulate TRIAP1 through hsa-miR-107. Drug sensitivity analysis was performed to explore the differences between the two risk groups. Among 198 drugs, 89 drug types exhibited significantly different estimated IC50 values between the two risk groups. Notably, Cisplatin, Docetaxel, Entinostat, Fludarabine, Lapatinib, Nelarabine, Talazoparib, and Vorinostat showed better therapeutic efficacy for high-risk MM patients (**Figures 10D–K**), these drugs have already been studied in MM.

**Figure 11:**
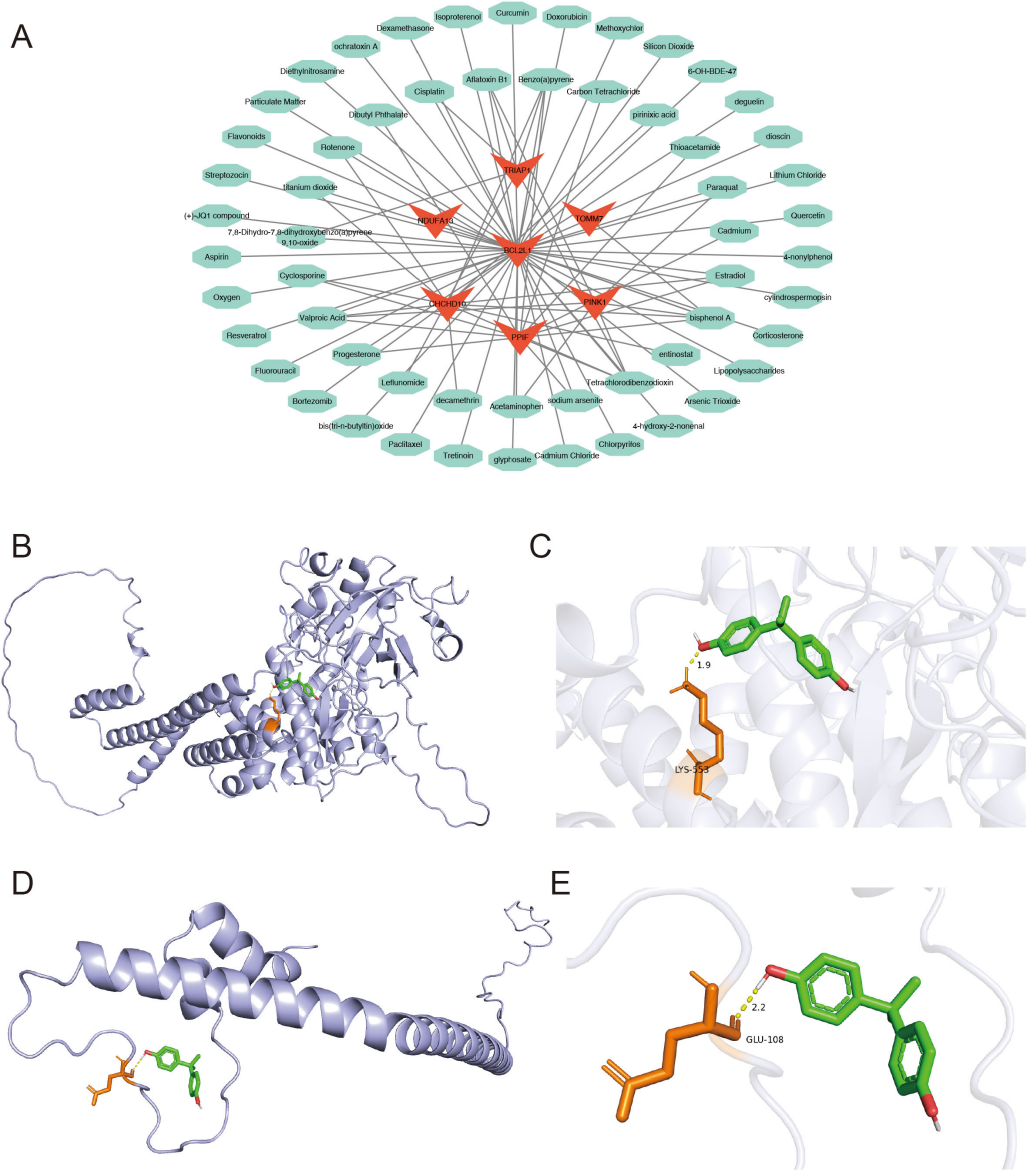
**(A)** Network diagram of prognostic gene-targeted compounds. **(B–E)** Molecular docking binding site diagrams: **(B, C)** PINK1 protein with Bisphenol A; **(D, E)** NDUFA13 protein with Bisphenol A.

Finally, 57 drugs were predicted to interact with the prognostic genes, and a drug-prognostic gene network was constructed comprising 64 nodes and 85 relational pairs. Notably, Bisphenol A was simultaneously predicted to interact with six prognostic genes (**Figure 11A**). Therefore, molecular docking simulations were performed to investigate the binding interactions between Bisphenol A and the prognostic genes. The binding free energy of Bisphenol A with PINK1 and NDUFA13 was less than -5 kcal/mol (**Table 1**), indicating favorable binding interactions. The hydrogen bonding sites between PINK1 and Bisphenol A were identified at LYS553 (**Figures 11B, C**), while the hydrogen bonding site between NDUFA13 and Bisphenol A was GLU108 (**Figures 11D, E**).

**Table 1:** Hub molecule dock.

PDB	Molecule Name	PubChem CID	Affinity(kcal/mol)	Hydrogen bonds
8AG0	Bisphenol A	6623	-4.68	1
AF-Q9P0U1-F1			-3.99	1
AF-Q9BXM7-F1			-5.04	1
AF-Q8WYQ3-F1			-4.14	0
7TH6			-4.8	1
1R2D			-4.52	1
AF-Q9P0J0-F1			-5.13	1

### 3.8 Prognosis genes had different expression in MM and control groups

**Figure 12:**
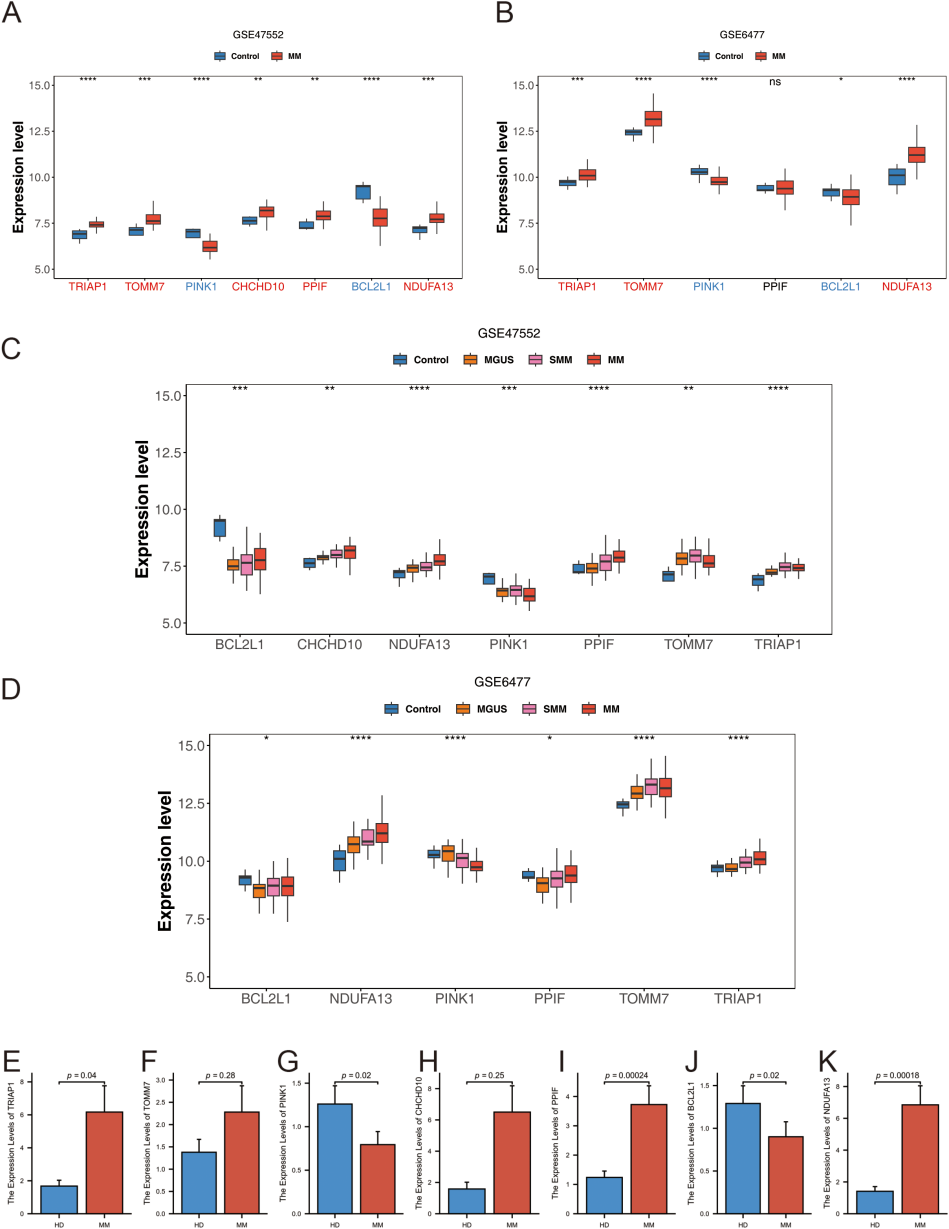
**(A, B)** Expression level of prognostic genes: **(A)** GSE47552; **(B)** GSE6477. **(C, D)** Expression levels of prognostic genes at different stages of plasma cell diseases: **(C)** GSE47552; **(D)** GSE6477. **(E–K)** The mRNA expression of TRIAP1 **(E)**, TOMM7 **(F)**, PINK1 **(G)**, CHCHD10 **(H)**, PPIF **(I)**, BCL2L1 **(J)** and NDUFA13 **(K)** by qRT-PCR in primary bone marrow mononuclear cells from MM and normal donors. Statistical significance is indicated as follows: *p < 0.05, **p < 0.01, ***p < 0.001, ****p < 0.0001, ns= no statistical significance.

The expression levels of the identified prognostic genes were analyzed by intergroup wilcoxon rank sum test in the GSE47552 (**Figure 12A**) and GSE6477 (**Figure 12B**) datasets. Among the six prognostic genes, the expression trends of *TRIAP1*, *TOMM7*, *PINK1*, *BCL2L1*, and *NDUFA13* in the validation set were consistent and significantly different from those in the training set, and the expression trend of PPIF gene in the validation set was not significant, but the expression trend was also consistent with the training set. Subsequently, we verified the expression trends of the seven prognostic genes in different subtypes. The results showed that six prognostic genes were all significantly different between different fractions in the training and validation sets (**Figures 12C, D**).

To further investigate the expression patterns of the prognostic genes, the single cell GSE151310 dataset, which comprehensively covers various cell types, was utilized. Four cell populations (CD4Tconv, Tprolif, CD8T, and CD8Tex) were annotated (**Supplementary Figures 3A, B**). The distribution of the prognostic genes across different cell clusters is shown in **Supplementary Figures 3C–I**. Notably, the expression levels of *TRIAP1, CHCHD10, TOMM7*, and *NDUFA13* were significantly elevated in MM samples, while *PINK1* and *BCL2L1* exhibited conspicuously reduced expression levels in MM.

To validate these findings in a clinical setting, samples were collected from both MM patient’s bone marrow and healthy donors (HD), and quantitative real-time polymerase chain reaction (qRT-PCR) was performed. Our observations indicated that the mRNA expression levels of *TRIAP1, TOMM7, CHCHD10, PPIF*, and *NDUFA13* were significantly elevated in MM patients compared to healthy donors (**Figures 12E, F, H, I, K**). Conversely, the mRNA expression of *PINK1* and *BCL2L1* were downregulated in MM patients (**Figures 12G, J**). Although the mRNA expression of *TOMM7* and *CHCHD10* in MM were not statistically significant, potentially due to a limited sample size, the overall trend was consistent with the bioinformatics analyses.

## 4 Discussion

MM is a malignant hematological malignancy characterized by uncontrolled plasma cell proliferation, lacking a definitive cure and posing a severe threat to patients’ lives. Mitochondria play a pivotal role in MM cell survival, proliferation, and drug resistance (42–46). PCD, encompassing apoptosis, necrosis, and autophagy, is crucial for maintaining homeostasis and is dysregulated in MM progression (47, 48). Mitochondrial dysfunction can induce cellular stress and initiate PCD pathways (49). To elucidate the pathogenesis of MM, this study delved into the roles of mitochondria and programmed cell death-related genes in multiple myeloma, identifying seven key prognostic genes correlated with patient outcomes, lower immune cell infiltration and altered immune function in the high-risk group, potential immune checkpoints and therapeutic drugs, regulatory miRNAs and lncRNAs, and validated the prognostic utility of the identified genes in clinical samples, providing novel insights into MM pathogenesis and evidence for prognostic assessment and treatment strategies.

We identified the DEGs in MM that overlapped with MRGs and PCDRGs. Prognostic analysis revealed seven key genes: *TRIAP1, TOMM7, PINK1, CHCHD10, PPIF, BCL2L1*, *NDUFA13*, and their chromosomal locations were determined. *TRIAP1*, a small anti-apoptotic protein, is vital for mitochondrial protection. It interacts with TP53 (50), a known regulator of apoptosis, implying that *TRIAP1* is involved in the regulation of cell death. In MM cells, *TRIAP1* may influence cell survival and proliferation by modulating related signaling pathways. The *PINK1* gene encodes a mitochondrial serine/threonine protein kinase, and previous studies in MM cells have shown that *PINK1*-dependent mitophagy plays a key role in regulating myeloma migration, homing, and tumorigenesis (51, 52). The protein encoded by the *TOMM7* gene is an integral component of the mitochondrial outer membrane transporter complex, essential for mitochondrial protein transport and assembly. While the specific role of *TOMM7* in MM remains elusive, mitochondrial protein transport is necessary to maintain mitochondrial function, thereby affecting energy metabolism and survival of MM cells. *CHCHD10*, located on the mitochondrial inner membrane, plays a pivotal role in mitochondrial function maintenance, including mitochondrial DNA stability and respiratory chain complex assembly. In MM, an abnormal elevation of *CHCHD10* may prevent mitochondrial destruction in MM cells and promote disease onset and progression. *PPIF* is a peptidyl-prolyl cis-trans isomerase located on the mitochondrial inner membrane that influences apoptotic signaling transmission. The *BCL2L1* gene belongs to the bcl-2 protein family, and its encoded protein, located on the mitochondrial outer membrane and regulates apoptosis. In MM, abnormally low expression of *BCL2L1* may lead to apoptosis resistance and disease progression (23, 53). The *NDUFA13* gene encodes a subunit of mitochondrial complex I, which is crucial for mitochondrial energy metabolism. *NDUFA13* may affect energy metabolism in tumor cells, particularly adapting to mitochondrial dysfunction and oxidative stress (54). Additionally, our research also found significant differences in 7 prognosis genes in MM, and how they affect prognosis.

The risk score model based on seven mitochondrial and PCD-related genes exhibited stable prognostic stratification across three independent datasets. KM analysis revealed significant survival differences across clinical subgroups. While initial AUC values around 0.6 indicated potential for improvement, the model’s performance could be enhanced through incorporating additional relevant features, optimizing hyperparameters, increasing sample size and diversity, controlling for confounders, and integrating ensemble learning methods. Notably, higher risk scores associated with advanced ISS stages, highlighting broad clinical applicability. Multivariate analysis confirmed the risk score as an independent prognostic factor. Notably, the nomogram achieved AUC values exceeding 0.7 at 1, 2, and 3 years, outperforming ISS staging in predicting patient prognosis. Functional enrichment analysis indicated higher activity in key biological processes like cell cycle, p53 signaling, and DNA replication in the high-risk group compared to the low-risk group. Previous study showed that, cell cycle (55, 56), p53 signaling pathway (57), and DNA replication (58) may play a crucial role in promoting the development of MM. As a therapeutic strategy for multiple myeloma, the nongenotoxic activation of the p53 pathway had potential application value (59). Therefore, the enriched pathways identified in the high-risk group may hold significant importance in MM pathogenesis and therapeutic targeting.

The role of the immune microenvironment in cancer is becoming increasingly important; therefore, this study explored the differences in immune cells and immune functions between multiple MM and the control group. We also conducted correlational analyses to identify cells significantly associated with prognostic genes and risk scores. Previous research showed immunosuppression in MM patients, manifested by increased CD8+ T cell exhaustion marker levels, hindering immunotherapy efficacy (60). γδ T cells exhibit potential antitumor effects in MM, and bisphosphonates that stimulate them may contribute to reduced plasma cell survival (61). Study showed that there was a bidirectional interaction between macrophages and MM tumor cells (62). Targeting bone marrow resident macrophages as a potential new therapeutic strategy for identified and recurrent multiple myeloma (63). Our study revealed significant differences in immune cell composition between high- and low-risk groups, with reduced infiltration of activated B cells, effector memory CD8+ T cells, γδ T cells, and macrophages in the high-risk group, consistent with previous findings and associated with poor prognosis. Significant differences were observed in immune functions like APC co-inhibition and checkpoints. Genetic interactions correlated with prognostic genes, and TIDE and ESTIMATE analyses suggested higher tumor immune evasion probability in the high-risk group, providing a rationale for immunotherapy. Notably, expression differences in immune checkpoint genes like CD48 (64), CD70 (65), and CTLA4 (66) offer potential intervention targets for future immunotherapy.

When exploring the regulatory mechanisms of MM, we also conducted an in-depth analysis of miRNAs and lncRNAs. The results indicated that several molecules, including hsa-miR-107, may play crucial roles in regulating the fate of MM cells (67, 68), although their specific functions require further investigation. Additionally, based on the characteristics of prognostic genes, we predicted potential therapeutic drugs for MM, some of these drugs have already been studied in MM. Cisplatin is used with other drugs, not alone, for MM treatment (69). In multiple myeloma with renal dysfunction, low-dose fludarabine/cyclophosphamide enabled successful idecabtagene vicleucel CAR T-cell therapy and complete remission (70). Preclinically, PARP inhibitors like olaparib and talazoparib synergized with melphalan against MM (71). FDA-approved histone deacetylase inhibitors vorinostat, belinostat, and romidepsin disrupt tumor growth processes, showing significant anti-MM activity (72). Our study found Cisplatin (73, 74), Entinostat (75), Fludarabine (76, 77), Talazoparib (71) and Vorinostat (78, 79) more effective in high-risk MM, potentially targeting pathophysiology. Cisplatin and docetaxel may inhibit tumor cell proliferation (80), while entinostat enhances anti-MM activity when combined with proteasome inhibitors (81). Fifty-seven drugs were predicted to interact with the prognostic genes, and molecular docking simulations revealed favorable binding of Bisphenol A with *PINK1* and *NDUFA13*, mediated by specific hydrogen bonding sites. These findings support future drug development. Last but not least, qPCR in clinical samples from MM patients and healthy donors validated the expression levels of the seven key prognostic genes, corroborating the bioinformatic findings.

In summary, this study utilized public transcriptomic data and provided novel insights into MM through systematic bioinformatic analyses. We identified prognostic mitochondrial and programmed cell death-related genes in MM and comprehensively explored the tumor immune microenvironment and immunotherapeutic potential. Future research should focus on functional studies to elucidate the specific mechanisms and clinical applications of these prognostic genes in MM.

## 5 Conclusion

This study conducted delved into the roles of mitochondria and PCD-related genes in MM. We successfully identified seven key prognostic genes whose expression patterns were closely correlated with patient outcomes. Furthermore, our analysis indicated lower level of immune cell infiltration in the tumor microenvironment of the high-risk patient group, along with variations in immune function. Additionally, we uncovered several potential immune checkpoints and therapeutic drugs, paving the way for future treatment approaches. Through the analysis of miRNAs and lncRNAs, we also revealed key molecules that may be involved in the regulation of MM. Importantly, we validated the prognostic utility of the identified genes in clinical samples. Overall, this study provides novel insights into the pathogenesis of MM and offers valuable scientific evidence for prognostic assessment and treatment strategies.

## Data Availability

The original contributions presented in the study are included in the article/**Supplementary Material**. Further inquiries can be directed to the corresponding author.
