# Supplementary figures and images for: Mitochondria-associated programmed cell death: elucidating prognostic biomarkers, immune checkpoints, and therapeutic avenues in multiple myeloma
Source: Front Immunol. 2024 Dec 11;15:1448764. doi: 10.3389/fimmu.2024.1448764 (PMC11670199; doi:10.3389/fimmu.2024.1448764)

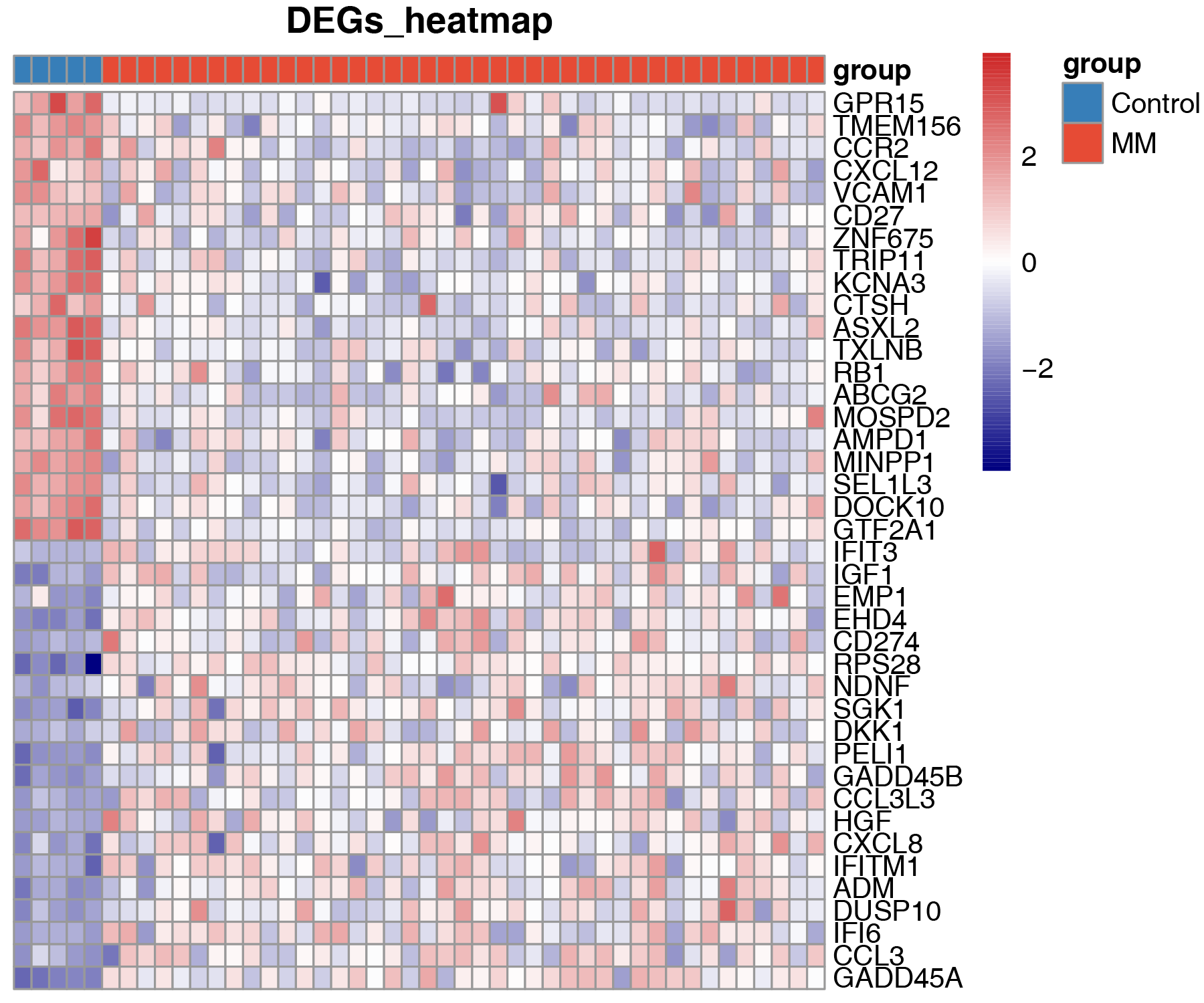

Supplement: Supplementary file 1 [file Image1.tif]

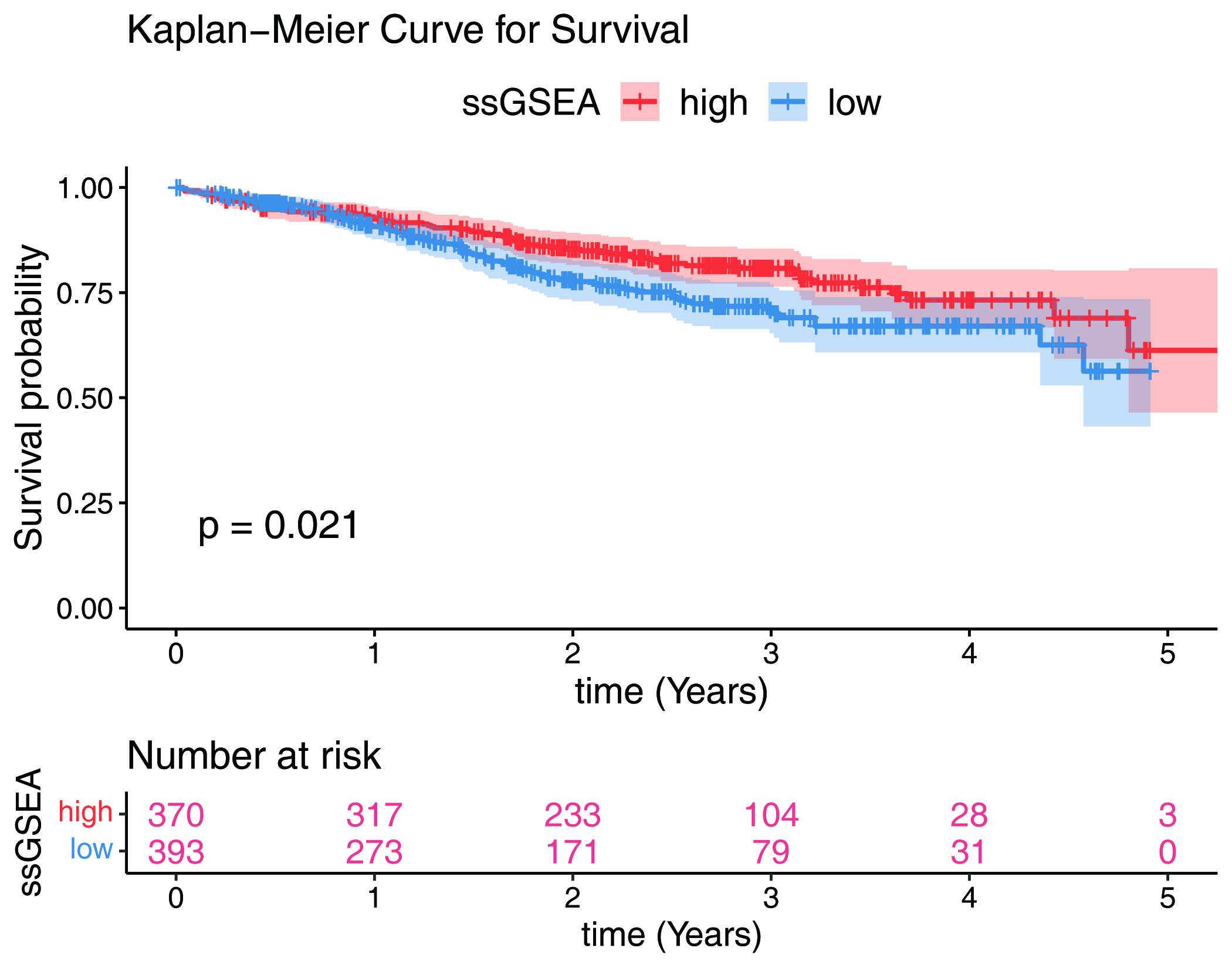

Supplement: Supplementary file 2 [file Image2.tif]

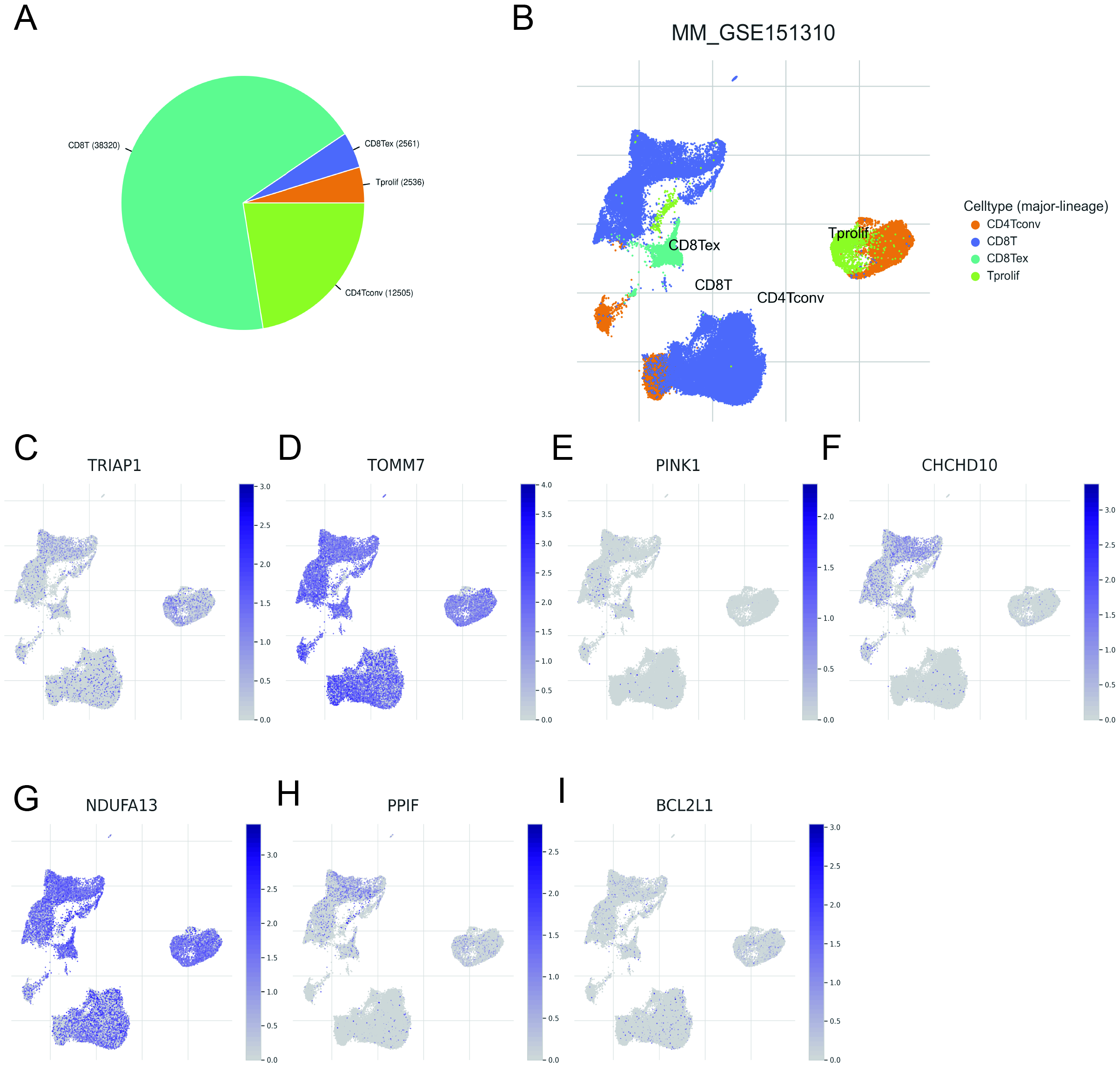

Supplement: Supplementary file 3 [file Image3.tif]
